# Fictitious Catalogues of Medical Students

**Published:** 1846-02

**Authors:** 


*Fictitious Catalogues of Medical Students.*—A correspondent in one of our large cities, calls our attention to a new subject, by saying that the profession is not aware, perhaps, "that certain medical schools in the United States are in the habit of issuing annually, a list or catalogue of students, far exceedin the *real number* of their matriculants or attendants. This is done for the purpose, no doubt, of swelling the importance of their institutions abroad, or in distant places, and with the view of attracting additional students the next year to their benches, under the idea that most young men like to congregate. Besides the fictitious names thus introduced into catalogues, the professors, or their agents," the writer says he is informed, "invite the apprentices of shomakers, tailors, carpenters and apothecaries, to attend their lectures, and add *these*, also, with some change of surname, &c., to their list of regular attendants. The whole of this fraudulent system is not only derogatory to those concerned in it, but injurious to the profession; inasmuch as it induces medical men in remote places, unaware of the imposition, to get up other medical schools, under the persuasion that they also can form classes with the same facility that Drs. A, B, and C have done in Philadelphia, New York, Baltimore, and Boston." The same gentleman says, further, that we shall "confer a favor upon many respectable physicians, by calling the attention of the profession to this subject at the present moment," and assures us that he "will undertake to prove his assertions a few weeks hence, by sending us the catalogues of such institutions as practice this foul iniquity, and by reviewing them and furnishing the names of their decoy ducks, with extended and suitable comments upon such transactions."—*Boston Med. & Surg. Jour.*

